# DISSOCIATING RETEST EFFECTS FROM DEVELOPMENTAL CHANGE FOR PREDICTING COGNITIVE STATUS

**DOI:** 10.1093/geroni/igac059.2410

**Published:** 2022-12-20

**Authors:** Nicholas Tamburri, Cynthia McDowell, Stuart MacDonald

**Affiliations:** University of Victoria, Montreal, Quebec, Canada; University of Victoria, Victoria, British Columbia, Canada; University of Victoria, Victoria, British Columbia, Canada

## Abstract

In longitudinal designs, unadjusted retest effects can confound developmental change estimates. This study utilized a measurement burst design and three-level multilevel modeling to a) independently parameterize short-term retest and long-term developmental change and b) employ these estimates as predictors of cognitive status at long-term follow-ups. Using data from Project MIND , participants (N=304; aged 64-92 years) were assessed across biweekly sessions nested within annual bursts (spanning up to 17 total assessments over four years). Cognitive impairment no dementia (CIND) status was classified at Years 4 (the final burst assessment) and 8 (the study end date). Response time inconsistencies (RTI) were computed to index intraindividual variability across RT trials of a one-back response time (BRT) task. Three-level multilevel models simultaneously yet independently estimated BRT RTI change across weeks and years, indexing short-term retest and long-term developmental change, respectively. Individual slope estimates were extracted and utilized in multinomial logistic regression models contrasting short- vs. long-term RTI change as predictors of long-term cognitive status. Results from the three-level models indicated that retest and developmental slopes yielded non-redundant sources of variance, providing unique estimates of change that would otherwise be confounded. Further, short- and long-term RTI differentially predicted cognitive status at Years 4 and 8; failing to benefit from retest effects on the BRT task was associated with increased likelihood of cognitive impairment. This innovative approach to parameterizing retest effects can reduce systematic bias in estimates of long-term developmental change, as well as highlight the utility of retest effects as predictors of cognitive health.

